# Editorial: The experiences of mental health professionals in psychiatric settings

**DOI:** 10.3389/fpsyt.2024.1416671

**Published:** 2024-05-23

**Authors:** Renato de Filippis, Maiko Fukasawa, Mohammadreza Shalbafan

**Affiliations:** ^1^ Psychiatry Unit, Department of Health Sciences, University Magna Graecia of Catanzaro, Catanzaro, Italy; ^2^ Health Promotion Center, Fukushima Medical University, Fukushima, Japan; ^3^ Mental Health Research Center, Psychosocial Health Research Institute, Department of Psychiatry, School of Medicine, Iran University of Medical Sciences, Tehran, Iran

**Keywords:** burnout, mental health professionals, moral injury, psychiatric care, well-being

Mental health professionals working in psychiatric settings face various stressors in their roles, which can negatively impact their physical, mental, and emotional well-being (1, 2). During crises like the COVID-19 pandemic or other challenging conditions, these effects can worsen, increasing the risks of stress, burnout, moral injury, and mental health issues among healthcare workers (3, 4). Despite these recognized challenges, there has been limited attention in the literature to promoting the health and well-being of mental health professionals (5). Indeed, their experiences in psychiatric centers, facilities, and hospitals remain relatively unexplored and little data is available (6). Of relevance is the topic of the occupational exposure to suicide, since it significantly contributes to suicidality, particularly among mental health professionals and first responders during their duties (7). For the purpose of this research topic, we have included in the definition of mental health professional each health care practitioner or social and human services provider who offers services for the purpose of improving an individual’s mental health or to treat mental disorders, therefore including students/trainees or residents, paramedical professionals (e.g., housing staff), as well as psychiatrists and psychologists.

Therefore, in this Research Topic, we aimed to collect articles dealing with the firsthand experiences, difficulties, frustration, and motivation of mental health professionals in psychiatric and psychological settings. Our goal has been to identify potential risk factors and effective strategies for supporting the mental health, as well as to improve their work, of these professionals through self-care and evidence-based interventions. Additionally, we sought to gather evidence highlighting the necessity for organizational measures, policies, and systemic changes. We finally included five papers dealing with this theme from different viewpoints.

Bloemendaal et al. presented the results of the CRITical Incidents and aggression in Caregivers (CRITIC) Study, which investigates the impact of childhood adversity and benevolence on the professional quality of life (ProQOL) of frontline psychiatry staff. This cross-sectional survey involves 360 participants from clinical and forensic psychiatry, exploring their personal history, trauma exposure, mental health, coping strategies, and work engagement. By examining how childhood experiences moderate the effects of workplace trauma on ProQOL, the study aims to understand and potentially enhance resilience among healthcare workers. The findings could inform interventions aimed at improving the well-being of healthcare professionals.

Thakur et al. conducted a qualitative analysis study about the implementation of an evidence-based four-phased feedback model (i.e., R2C2) in a psychiatry competency-based medical education residency program, using the Consolidated Framework for Implementation Research (CFIR). The interest of this study lies in the possibility of having structured feedback on the work of mental health professionals, with the aim of improving the service and making it more functional for patients. Fifteen supervisors, after training, utilized the R2C2 model with residents. Through semi-structured interviews with 10 supervisors, the study explores their experiences with the model. Qualitative analysis reveals four main themes: perceptions of the R2C2 model, facilitators and barriers to its implementation, fidelity to the model, and intersectionality regarding feedback. The R2C2 model proves beneficial for structured feedback. Key facilitators of implementation include the model’s structure, self-efficacy, educational expertise, learning culture, organizational readiness, and training support.

Durif-Bruckert et al., with the first qualitative study based on focus groups on the impact of patient suicide on psychiatric residents, explored the impact of this exposure on French psychiatric residents’ careers and practical experiences. Nineteen residents participated in focus groups, and, through content analysis, four thematic clusters emerged: reactions to exposure, coping strategies, professional impact, and proposals for prevention and postvention. Participants emphasized the crucial role of support, particularly from senior staff, in mitigating negative effects. They also offered suggestions to enhance prevention and postvention efforts, and the findings highlight the importance of support mechanisms and suggest strategies to minimize its adverse effects.

In the paper by Molnár et al., authors examine stress, burnout, and sleeping difficulties among mental health professionals in Hungary, particularly those involved in COVID care during the fourth and fifth waves of the pandemic, through an online cross-sectional survey involving the Copenhagen Psychosocial Questionnaire (COPSOQ II). Participation in COVID care correlated with heightened work pace, increased role conflicts, decreased influence at work, predictability, reward, role clarity, social support from supervisors, job satisfaction, trust in management, justice, and respect. Moreover, competence transgression significantly affected stress levels, and being a psychiatric specialist contributed to higher burnout. These factors collectively explained a significant portion of the variance in stress and burnout levels. Despite improved organization of COVID care during subsequent waves, psychiatrists faced challenges regarding their competence and influence at work, likely contributing to their heightened stress and burnout levels.

Finally, Brolin et al. tried to better understand the perspectives of housing staff on stimulating and supporting resident engagement in activities. Indeed, housing staff may often convey their frustration regarding offering such support to encourage residents to participate in meaningful activities, as well as the challenge of determining appropriate levels of independence within a housing environment constrained by limitations. Therefore, twenty-six staff members from 20 supported housing units across Sweden participated in five focus groups. Through semi-structured interviews and qualitative content analysis, three main categories emerged: factors influencing activity support provision, staff approaches to supporting activities, and staff’s struggles in their work development. The analysis revealed various obstacles to community activity participation and highlighted conflicting factors like spontaneity versus structure and individual versus group activities, impacting staff motivation efforts. To enhance resident activity engagement within Supported Housing, a comprehensive approach is suggested, including in-house training focusing on values, recruitment policies, staff supervision, and interventions targeting both residents and staff. This approach aims to support staff in effectively motivating residents towards increased activity participation.

In conclusion, in this research topic we analyzed experiences of mental health professionals in psychiatric settings from five different viewpoint and perspectives. We welcomed all articles potentially able to add some elements of novelty and evidence on this sensitive topic.

The changes and improvements proposed by this research are essential for addressing the challenges faced by mental health workers and empowering them in their roles in the future.

## Author contributions

RF: Writing – original draft. MF: Writing – review & editing. MS: Writing – review & editing.
